# Beurteilung der Betreuung dementer Patienten im allgemeinärztlichen Hausbesuch

**DOI:** 10.1007/s00391-020-01715-4

**Published:** 2020-03-18

**Authors:** Fabian Lenz, Jeannine Schübel, Robert Neumann, Antje Bergmann, Karen Voigt

**Affiliations:** 1 Lehrstuhl Allgemeinmedizin der Medizinischen Fakultät Carl Gustav Carus, TU Dresden, Fetscherstraße 74, 01307 Dresden, Deutschland; 2 Professur für Methoden der empirischen Sozialforschung am Institut für Soziologie, TU Dresden, Dresden, Deutschland

**Keywords:** Wohnort, Demenz, Versorgungsqualität, Pflegeheim, Allgemeinmedizin, Residence, Dementia, Quality of care, Nursing home, Family medicine

## Abstract

**Hintergrund:**

Demenzpatienten (DP) sind eine besondere Herausforderung für das Gesundheitssystem. Auf sie entfallen 5 % der Gesamtausgaben im Gesundheitswesen. Die krankheitsbedingten Defizite und der damit verbundene Versorgungsbedarf führen dazu, dass viele Patienten nicht mehr in der eigenen Häuslichkeit leben können und auf die Versorgung in Pflegeheimen (PH) angewiesen sind.

**Fragestellung:**

Wie wird die gesamtheitliche Betreuung von DP im Hausbesuch (HB) eingeschätzt?

Hat die regionale Lage Einfluss auf die Wohnsituation bei DP?

**Material und Methoden:**

Im Rahmen von SESAM‑5 wurden von 303 sächsischen Hausarztpraxen in einem Zeitraum von einem Jahr 4286 HB mittels Fragebogen dokumentiert und sowohl inhaltliche als auch strukturelle Merkmale erfasst.

**Ergebnisse:**

Die Prävalenz von Demenz bei HB-Patienten betrug 27,5 %. Von diesen bewohnten 72,6 % ein PH oder betreutes Wohnen. Das medizinische Personal schätzte die gesamtheitliche Betreuung von DP im PH signifikant besser ein als in der eigenen Häuslichkeit. Diese Diskrepanz fiel im Vergleich von ländlichen und städtischen Gebieten noch deutlicher aus, obwohl in städtischen Gebieten signifikant mehr Patienten in PH wohnten (27 % vs. 51 %).

**Diskussion:**

Die Betreuung dementer HB-Patienten wird vom versorgenden Personal überwiegend als gut eingeschätzt, wobei diese bei DP im PH vergleichsweise besser bewertet wird als in der eigenen Häuslichkeit. Dies kann durch einen hohen Versorgungsbedarf der DP erklärt werden. Der Kontrast zwischen ruralen und städtischen Gebieten ist durch infrastrukturelle, aber auch durch organisatorische Unterschiede in ländlichen Bereichen erklärbar, wo signifikant häufiger eine Mitbetreuung durch Angehörige erfolgt. Da bei DP kognitive Defizite im Vordergrund stehen, könnten alternative Wohnformen zukünftig mehr in den Fokus rücken.

## Abstract (en)

**Background:**

Patients with dementia (PD) are a special challenge for the healthcare system. They are responsible for 5% of the expenditure in the German healthcare service. The disease-related deficits and the associated need for care leads to the fact that patients are not able to live in their own residence and rely on the care of nursing homes (NH).

**Objective:**

How is the overall care in PD assessed in house calls (HC)? Does the regional situation influence the living conditions of PD?

**Material and methods:**

As part of the SESAM‑5 study 303 participating general practices in Saxony were asked to document their HC within a period of 1 year whereby 4286 HC were documented through questionnaires and analyzed for content and structural data.

**Results:**

The prevalence of dementia in HC patients was 27.5% and 72.6% of PD lived in a NH or assisted living home. The medical staff assessed the overall care of PD in the NH to be significantly better than in their own residence. This discrepancy was greater in rural compared to urban regions although in urban regions significantly more patients live in NHs (27% vs. 51%).

**Conclusions:**

The overall care of PD in HC was assessed predominantly as good by medical personnel, whereby PD in NH were assessed comparatively better than those in their own residence. This could be explained by the high need of care in PD. The difference between rural and urban regions is explainable through differences in the infrastructure and also in the organization in rural areas, where relatives participate in care significantly more frequently. In the future more focus could be placed on alternative types of housing because in PD cognitive deficits are in the foreground.

## Hintergrund und Fragestellung

Die Herausforderungen des demografischen Wandels in Deutschland sind aktueller denn je. Mit steigender Lebenserwartung steigt auch die Prävalenz chronischer Krankheiten. Dabei stellt eine Demenzdiagnose besondere Herausforderungen an Angehörige sowie medizinische Versorger. Nach Zahlen des Statistischen Bundesamts muss mit einem Anstieg der Prävalenz der Demenz bei über 65-Jährigen von 17 % auf bis zu 22 % in den nächsten 15 Jahren gerechnet werden. Das entspricht einer Zunahme an Demenzerkrankten in diesem Alter von 500.000 Patienten [4]. Dabei ist zusätzlich die große Zahl an nichtdiagnostizierten Patienten zu berücksichtigen [5]. Im Jahr 2015 wurden für die Versorgung und Behandlung von Patienten mit Demenz 15,1 Mrd. € ausgegeben; das sind 4,5 % der Gesamtausgaben im Gesundheitswesen [4]. Diese Kosten sind nicht nur in der rein medizinischen Behandlung begründet, sondern v. a. durch den zunehmenden Pflegebedarf, den die Patienten im Laufe ihrer Krankheit entwickeln [3]. Deshalb ist die Demenz auch ein Hauptrisikofaktor für einen Umzug ins Pflegeheim [6]. Es ist davon auszugehen, dass mehr als die Hälfte aller Pflegeheimbewohner an einer Demenz leiden [6, 7]. Jedoch auch die Patienten, die in der Häuslichkeit leben und häufig von ihren Angehörigen mitversorgt werden, brauchen zusätzliche pflegerische Unterstützung und eine adäquate hausärztliche Versorgung. Da der Besuch der Arztpraxis für die älteren Patienten im Kontext ihrer meist bestehenden Multimorbidität häufig große Herausforderungen darstellt, sind sie oft auf Hausbesuche angewiesen. Gerade Patienten in Pflegeheimen ist ein Besuch der Praxis meist nicht mehr möglich, sodass 96,9 % der Arztkontakte im Hausbesuch stattfinden [7]. Trotz des großen Bedarfs haben ca. 90 % der Pflegeheimbewohner monatlichen direkten Kontakt zu ihren Hausärzten [6, 7, 10]. Die vorliegende Studie beschreibt die Versorgungsqualität bei Hausbesuchspatienten mit Demenz in Abhängigkeit von Wohnsituation und geografischer Wohnlage.

Die Analyse fokussiert auf folgende Forschungsfragen:

1. Sind Patienten mit Demenz in einem Pflegeheim nach Einschätzung des medizinischen Personals qualitativ besser unterstützt als in der eigenen Häuslichkeit?
2. Gibt es regionale Einflüsse auf die bestehenden Versorgungsstrukturen und ggf. auch auf die Versorgungsqualität?

## Studiendesign und Untersuchungsmethoden

Im Rahmen der 5. Sächsischen Epidemiologischen Studie in der Allgemeinmedizin (SESAM-5) wurden durch den Bereich Allgemeinmedizin der TU Dresden in Kooperation mit der Sächsischen Gesellschaft für Allgemeinmedizin (SGAM) alle Hausarztpraxen in Sachsen um die Dokumentation ihrer Hausbesuche anhand eines standardisierten Fragebogens gebeten. Die Methodik ist detailliert im publizierten Studienprotokoll beschrieben [14]. Von 303 teilnehmenden Praxen wurden in einem Zeitraum von einem Jahr (Juli 2014 bis Juni 2015) 4286 Hausbesuche dokumentiert. Jede Praxis erhob die Hausbesuche innerhalb einer randomisiert zugewiesenen Woche, um saisonale Unterschiede auszugleichen. Zusätzlich zu den Merkmalen der Hausbesuche wurden auch Informationen über die Praxis selbst, wie Lage, Hausbesuchsstruktur und Patientenzahlen, erfasst. Die Betreuungsqualität der Patienten wurde von den Durchführenden des Hausbesuchs mittels Schulnoten bewertet („Wie schätzen Sie die gesamtheitliche Betreuung des Patienten ein?“), und an der Betreuung Beteiligte konnten erfasst werden. Die Daten wurden mit SPSS 25 (IBM, Armonk, NY, USA) und Stata 13 (StataCorp, College Station, TX, USA) ausgewertet und hinsichtlich der Grunderkrankung Demenz gruppiert. Dafür wurden die ICD-10-Codes F00 bis F03 herangezogen. Des Weiteren wurden die 5 meist dokumentierten Dauerdiagnosen in der Kohorte als Vergleichsgruppen herangezogen. Bei der statistischen Analyse wurde auf nichtparametrische Tests zurückgegriffen, da keine Normalverteilung vorlag. Ergänzend zu bivariaten Testungen wurde ein arztzentriertes Mehrebenenmodell auf Basis einer linearen Regression erstellt.

## Ergebnisse

### Beschreibung der Studienkohorte

Von 4286 besuchten Patienten waren 66,4 % (*n* = 2848) weiblich. Das Durchschnittsalter betrug 82,3 (Standardabweichung [SD] ±11,1) Jahre. 66 % (*n* = 2829) der Patienten hatten bereits mindestens Pflegestufe 1 (zum Zeitpunkt der Erhebung erfolgte die Einteilung nach Pflegestufen, die mittlerweile durch die Pflegegrade ersetzt wurde). Bei den besuchten Patienten wurden im Mittel 6,5 (SD ±4,0) Dauerdiagnosen dokumentiert. Der Großteil wohnte in der eigenen Häuslichkeit (58,2 %; *n* = 2436). Dem gegenüber waren mehr als ein Drittel der Hausbesuchspatienten in einem Pflegeheim oder im betreuten Wohnen untergebracht (41,8 %, *n* = 1749). 97 % (*n* = 4162) erfuhren Unterstützung durch Angehörige oder Pflegepersonal. Die Qualität dieser Unterstützung wurde durch das medizinische Personal (auch durchführende medizinische Fachangestellte [MFA]) im Hausbesuch als meist befriedigend bis sehr gut eingeschätzt (95 %, *n* = 3964) (Tab. 1).

**Table 1:** 

	Gesamt (*n* = 4286)		Demenz (*n* = 1180)		Keine Demenz (*n* = 3106)	
	Mittelwert	SD	Mittelwert	SD	Mittelwert	SD
*Alter*	82,3	±11,1	86,0	±7,5	80,9	±11,8
*Anzahl, Dauerdiagnosen*	6,5	±4,0	6,7	±4,0	6,4	±3,9
*Bewertung der Betreuung*^*a*^	1,71	±1,0	1,64	±0,9	1,74	±1,0
	Anteil (%)	*n*	Anteil (%)	*n*	Anteil (%)	*n*
*Geschlecht*						
Weiblich	66,4	2848	73,2	856	64,9	1992
Männlich	32,4	1389	26,8	314	35,1	1075
*Pflegestufe min. Stufe 1*	66,0	2829	83,9	990	59,2	1839
*Pflegeheimbewohner*	35,6	1525	62,6	721	26,5	804
*Bewohnen der eigenen Häuslichkeit*	57,0	1436	32,3	372	68,0	2064

^a^Bewertung nach Schulnoten (1: sehr gut, 6: ungenügend)

(Anmerkung: Fehlende Anteile ergeben sich durch Fallausschluss bei unvollständigen Angaben)

### Beschreibung der Teilstichprobe Patienten mit Demenz

Die Prävalenz von Demenz in der Studienkohorte betrug 27,5 % (*n* = 1180). Bei Bewohnern von Pflegeheimen lag diese bei 47,3 % (*n* = 721). Nahezu drei Viertel (72,6 %; *n* = 779) der besuchten Patienten mit Demenz bewohnten ein Pflegeheim oder betreutes Wohnen. Weiterhin stieg der Anteil an weiblichen Patienten im Vergleich zur Gesamtkohorte signifikant an (*p* < 0,001; Chi-Quadrat-Test). Bei 83,9 % (*n* = 990) bestand ein Pflegebedarf mit bereits bestehender Pflegestufe. Die Wahrscheinlichkeit, dass eine Pflegestufe vorlag, war bei Demenzpatienten signifikant höher (*p* < 0,001; Chi-Quadrat-Test). So stieg der Anteil an Pflegestufe II von 20,7 % auf 38,3 % und an Stufe III von 5,9 % auf 18,6 %. Die Demenzpatienten waren im Durchschnitt auch signifikant älter (86,0 ± 7,5 SD (dement) vs. 80,9 ± 11,8 SD Jahre (nichtdement); *p* < 0,001; Mann-Whitney-U-Test) und vereinten signifikant mehr Nebendiagnosen auf sich (6,7 ± 4,0 SD (dement) vs. 6,4 ± 4,0 SD (nichtdement), *p* < 0,02; Mann-Whitney-U-Test) als Patienten ohne dokumentierte Demenzdiagnose.

### Bewertung der Betreuungssituation von Demenzpatienten

Von den 4286 durchgeführten Hausbesuchen wurden 81,7 % (*n* = 3503) von den Hausärzten selbst durchgeführt. Alternativ kamen in 5,2 % (*n* = 225) der Fälle nichtärztliches Personal und jeweils 3,1 % (*n* = 132) MFA mit Zusatzqualifikation (z. B. Versorgungsassistent in der Hausarztpraxis [VERAH]) oder Ärzte in Weiterbildung zum Einsatz. Das ärztliche und nichtärztliche Personal im Hausbesuch schätzte die gesamtheitliche Betreuungsqualität von Demenzpatienten signifikant besser ein (1,64 ± 0,9 SD vs. 1,74 ± 1,0 SD, *p* < 0,05; Mann-Whitney-Test) als bei Patienten ohne Demenzdiagnose. Es ist jedoch festzuhalten, dass es signifikante Unterschiede der Bewertung nach Wohnform (*p* < 0,001; Kruskal-Wallis-Test) gab: Während die Betreuung von Pflegeheimpatienten mit Demenz mit einer durchschnittlichen Schulnote von 1,5 (±0,7 SD) bewertet wurde, lag die durchschnittliche Bewertung bei in der Häuslichkeit lebenden Patienten nur bei 2,1 (±1,1 SD). Es fällt auf, dass die Betreuung in der Häuslichkeit tendenziell leicht schlechter eingeschätzt wird, wenn ambulante Pflegedienste beteiligt sind.

Weiterhin zeigt sich ein Einfluss der regionalen Lage. In der Großstadt wird die Betreuung von Demenzkranken in der Häuslichkeit mit 2,3 (±1,0 SD) bewertet, im Kleinstadtbereich mit 2,1 (±1,3 SD). Entgegengesetzt steigt mit der Einwohnerzahl auch der Prozentsatz an Pflegeheimpatienten signifikant an (*p* < 0,001; Chi-Quadrat-Test): In der Kleinstadt (unter 5000 Einwohner) wohnen 26,5 % (*n* = 314) der Hausbesuchspatienten im Pflegeheim, in der Großstadt (über 100.000 Einwohner) dagegen 50,9 % (*n* = 411). Bei den Demenzpatienten sind es 49,3 % (*n* = 151) in der Kleinstadt und 76,9 % (*n* = 160) in der Großstadt (Tab. 2).

**Table 2:** 

Patienten mit Demenz (*n* = 1143)	Kleinstadt (*n* = 306)		Großstadt (*n* = 208)	
Wohnsituation	Anteil	Bewertung^a^	Anteil	Bewertung^a^
Allein (*n* = 143)	14,7 % (*n* = 45)	2,1 (SD ±1,3)	9,6 % (*n* = 20)	2,3 (SD ±1,0)
Mit Familie (*n* = 228)	29,7 % (*n* = 91)	1,9 (SD ±1,2)	11,5 % (*n* = 24)	2,0 (SD ±1,0)
Pflegeheim (*n* = 715)	49,3 % (*n* = 151)	1,6 (SD ±1,1)	76,9 % (*n* = 160)	1,8 (SD ±0,6)

^a^Bewertung nach Schulnoten (1: sehr gut bis 6: ungenügend)

(Anmerkung: Fehlende Anteile ergeben sich durch Fallausschluss bei unvollständigen Angaben)

Als Merkmal der Hausbesuche bei dementen Patienten zeigte sich in der bivariaten Analyse eine kürzere Hausbesuchsdauer (13,0 ± 9,1 SD min vs. 15,4 ± 9,3 SD; *p* < 0,001; Mann-Whitney-U-Test). Es handelte sich bei Patienten mit Demenz häufiger um Routinebesuche (73,5 %; *n* = 864 vs. 70,1 %; *n* = 2155), und es wurden häufiger Folgetermine vereinbart (60 %; *n* = 665 vs. 53 %; *n* = 1530). Bei Demenzkranken wurden signifikant weniger Einweisungen (2,2 %; *n* = 25 vs. 3,4 %; *n* = 97) und Überweisungen (1,3 %; *n* = 15 vs. 2,2 %; *n* = 64) ausgestellt (jeweils *p* < 0,05; Chi-Quadrat-Test). Es ergab sich für die Dringlichkeit des Hausbesuchs (Routine- vs. Notfallbesuch) und die Bewertung der Betreuung kein signifikanter Unterschied in Abhängigkeit vom professionellem Hintergrund der Hausbesuchsdurchführenden.

Um den Einfluss der wichtigsten Parameter auf die Beurteilung der Betreuung in einem multivariaten Modell zu analysieren und gleichzeitig die Abhängigkeit der arztspezifischen Einschätzung zu berücksichtigen, wurde eine lineares Mehrebenenmodell mit arztspezifischen „intercepts“ erstellt (Tab. 3). Dabei wurden die Parameter über die Ärzte gemittelt, sodass interindividuelle Unterschiede im Bereich der Ärzteschaft herausgerechnet werden. Der Referenzpatient ist dabei der alleinlebende, nichtdemente Patient in einem Wohnort mit weniger als 5000 Einwohnern im Rahmen eines Routinehausbesuchs.

**Table 3:** 

	Leeres Modell	
Demenz	–	0,075^*^
(Ref.-Kat. nein)	–	(0,034)
5000–10.000	–	−0,092
(Ref.-Kat. <5000)	–	(0,111)
10.000–50.000	–	−0,197^*^
	–	(0,094)
50.000–100.000	–	−0,214
	–	(0,204)
>100.000	–	0,055
	–	(0,106)
Privathaushalt, mit Partner/Familie	–	−0,320^***^
(Ref.-Kat.: allein lebend)	–	(0,038)
Betreutes Wohnen	–	−0,306^***^
	–	(0,070)
Alten- oder Pflegeheim	–	−0,536^***^
	–	(0,041)
Alter	–	−0,491^***^
	–	(0,140)
Weibliche Patientin	–	−0,099
	–	(0,075)
Intercept	1,752^***^	2,182^***^
	(0,036)	(0,087)
Var (L2 Ärzte)	0,270^***^	0,256^***^
	(0,030)	(0,029)
Var (intercept)	0,742^***^	0,721^***^
	(0,017)	(0,017)
AIC	11.051,1	10.445,3
BIC	11.070,1	10.527,1
L1 N (Patienten)	4172	3978
L2 N (Ärzte)	253	247
Snijders/Bosker R^2^ (L1)	–	0,049
Snijders/Bosker R^2^ (L2)	–	0,054

Standardfehler in Klammern; ^*^p < 0,05, ^**^p < 0,01, ^***^p < 0,001

**Figure 1:**
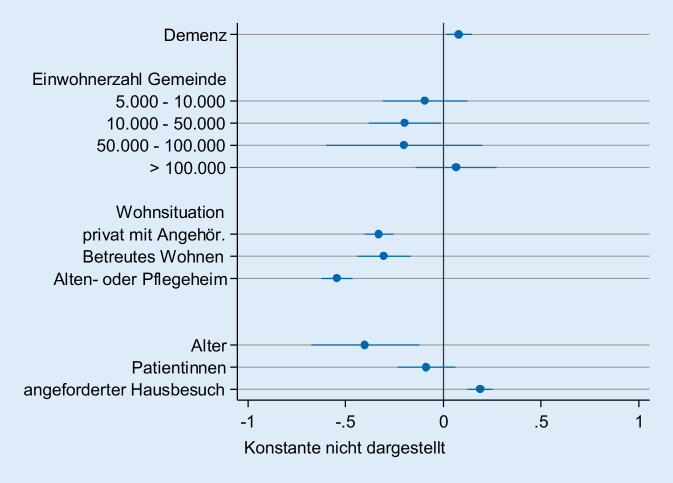

Es ergab sich ein Modell, in dem sich die Wohnsituation als stärkster Prädiktor präsentierte. Demenzpatienten sind dabei im Vergleich zum Referenzpatienten signifikant schlechter versorgt, ebenso Patienten in einem angeforderten Hausbesuch. Die Gemeindegröße und das Geschlecht der Patienten konnten die Ausprägung der Beurteilung der Betreuung nicht substanziell erklären. Das bedeutet, dass ein durchschnittlicher Arzt in einem angeforderten Hausbesuch bei einem dementen Patienten, der allein in der eigenen Häuslichkeit lebt, am ehesten dazu neigt, eine schlechte Bewertung für die Einschätzung der Unterstützung abzugeben. In Abb. 1 ist der Einfluss durch die verschiedenen Faktoren innerhalb des multivariaten Modells auf die Schulnote der Bewertung dargestellt.

### Einfluss von Demenz auf die Bewertung der Betreuung im Vergleich zu anderen häufigen Erkrankungen

Neben der Demenz wurden die 4 häufigsten Dauerdiagnosen bestimmt: Dies waren die arterielle Hypertonie (Prävalenz von 60 %), Diabetes mellitus (41 %), KHK (23,6 %) und Herzinsuffizienz (18,4 %). Dabei ergab sich in der bivariaten Analyse lediglich für die Demenz der bereits oben genannte signifikante Einfluss. Die anderen Dauerdiagnosen hatten keine signifikanten Auswirkungen.

## Diskussion

### Vergleichbarkeit der Studie

Das Durchschnittsalter der Patienten lag im Bereich vergleichbarer Studien zu Hausbesuchen [9, 11–13] bzw. Pflegeheimbewohnern. Der Anteil weiblicher Patienten lag etwas unter dem Durchschnitt von Patientinnen in vergleichbaren Demenz- und Pflegeheimstudien [6, 7]. Durch die höhere Lebenserwartung sind diese dort überrepräsentiert, während eine Hausbesuchsstudie einen breiteren Querschnitt abbildet.

Die Prävalenz von Demenz lag in der vorliegenden Studie niedriger als in vergleichbaren Studien (47,3 % in Pflegeheimen vs. 57 %/68 % [6, 7]). Es ist jedoch von einer Unterschätzung auszugehen, da in der Studie keine Diagnosekriterien erhoben, sondern lediglich bereits bestehende Dauerdiagnosen dokumentiert wurden. Die Anzahl an nichtdiagnostizierten Erkrankungen im Bereich der Demenz ist gerade bei Frühformen und im hausärztlichen Setting besonders hoch [15]. Die Prävalenzen anderer häufiger Dauerdiagnosen lagen erwartungsgemäß über den Prävalenzen der Gesamtbevölkerung, da Hausbesuche in der Regel eine ältere Patientenklientel abdecken.

### Einflussgröße Demenz

Die gesamtheitliche Beurteilung der Betreuung von dementen Patienten wird durch die Hausbesuchenden insgesamt als gut bis sehr gut beschrieben. Es fällt auf, dass die Patienten mit Demenz in den meisten Fällen durch Angehörige und Pflegedienste Unterstützung im Alltag erfahren oder in Pflegeheimen durch das dortige Personal unterstützt werden. Demenzkranke, die in der eigenen Häuslichkeit leben und keine Unterstützung erfahren, sind dagegen etwas schlechter versorgt. Die Betreuung von Patienten im Pflegeheim wird dabei etwas besser bewertet als bei in der privaten Häuslichkeit lebenden Patienten, was durch geringere professionelle Unterstützung erklärbar sein könnte. Dass Patienten, die durch einen ambulanten Pflegedienst unterstützt werden, als schlechter betreut eingeschätzt werden, könnte man am ehesten als Vorzeichen eines potenziell höheren Pflegebedarfs werten.

Im bivariaten Vergleich mit anderen häufigen Dauerdiagnosen zeigt sich die Demenz als einziger signifikanter Einflussfaktor auf die Bewertung der Betreuung. Demente Patienten sind dabei signifikant häufiger in Heimen untergebracht und haben eine höhere Pflegestufe. Die subjektive Einschätzung der Betreuung bei Demenzpatienten fällt in der bivariaten Testung ebenfalls signifikant schlechter aus, wenn sie keine professionelle Unterstützung erhalten.

Insgesamt bestätigen die Ergebnisse den großen Bedarf an Unterstützung bei Patienten mit Demenzdiagnose. Während andere Dauerdiagnosen unter Mithilfe von Angehörigen oder Pflegediensten ambulant führbar sind, erfordert es bei Patienten mit Demenz eine noch umfassendere Betreuung, die häufig nur durch Unterbringung im Pflegeheim gewährleistet werden kann. Findet jedoch im Voraus eine vollumfängliche Beratung über zusätzliche Unterstützungsleistungen statt und werden diese auch umgesetzt, kann der Umzug ins Pflegeheim hinausgezögert werden [2].

### Einflussgröße Stadt/Land

Die Annahme, dass sowohl die Wohnform als auch die Gemeindegröße die maßgeblichen Prädiktoren für die Betreuungsqualität bei Demenzpatienten sind, konnte nicht eindeutig bestätigt werden. Insbesondere für die Gemeindegröße ergaben sich uneinheitliche Befunde. Im Mehrebenenmodell wird aber deutlich, dass v. a. die Wohnform starken Einfluss hat. Es konnte aber auch ein Zusammenhang zwischen Gemeindegröße und Wohnsituation beobachtet werden. Während in ländlichen Gebieten Demenzkranke häufiger in der eigenen Häuslichkeit betreut werden, findet in der Großstadt eine weitestgehende Betreuung in Pflegeheimen statt. Die Qualität wird im ländlichen Raum jedoch auch bei Versorgung in der Häuslichkeit besser eingeschätzt als in der Stadt. Es bleibt offen, ob dies strukturelle oder organisatorische Gründe hat. Die gute Versorgung auf dem Land lässt annehmen, dass die familiären Strukturen (z. B. Mehrgenerationenwohnform) es eher ermöglichen, demente Patienten auch in der Häuslichkeit adäquat zu versorgen, während in den Städten der Trend zu Einzelhaushalten steigt [1]. Alternativ wäre auch möglich, dass fehlende Pflegeheime keine andere Versorgung zulassen und damit auch unter Einbeziehung der Hausärzte bessere informelle Hilfsstrukturen entstanden sind.

Im Hinblick auf eine zunehmende Urbanisierung und Engpässe im Bereich der Pflege müssen alternative Wohnformen diskutiert werden: Da oft die kognitiven Einschränkungen im Vordergrund stehen, besteht v. a. Bedarf im Bereich der Betreuung und weniger im Bereich der Pflege. So werden voraussichtlich Formen des betreuten Wohnens oder betreute Demenz-WG sowie entlastende ambulante Angebote (z. B. Tagespflege) zunehmend in den Fokus des öffentlichen Interesses rücken [8].

### Limitierung

Es ist zu berücksichtigen, dass es sich in der vorliegenden Studie um die Erfassung von Hausbesuchspatienten handelt, die im Vergleich zur durchschnittlichen Klientel einer Hausarztpraxis häufig im Alltag stärker eingeschränkt sind. Die Bewertung der Betreuung von Patienten, die möglicherweise gar keine Arztkontakte oder nur in der Sprechstunde haben, wird nicht erfasst. Ohnehin muss von einer hohen Zahl nichtdiagnostizierter Demenzen gerade im Bereich alleinlebender Patienten ausgegangen werden. Bei der Einschätzung der Betreuung handelt es sich um eine subjektive Beurteilung der medizinischen Versorger. Die teils geringeren Unterschiede sollen dabei als Hinweis auf ein Verbesserungspotenzial gesehen werden. Für eine objektive Vergleichbarkeit müsste die Versorgung anhand harter Kriterien, z. B. Qualitätsindikatoren, eingeordnet werden.

## Schlussfolgerung und Fazit für die Praxis

- Die Betreuung dementer Hausbesuchspatienten im Alltag wird vom betreuenden ärztlichen und nichtärztlichen Personal überwiegend als gut eingeschätzt.
- Patienten mit Demenzdiagnose sind nach subjektiver Einschätzung des durchführenden Personals im Pflegeheim vergleichsweise besser versorgt als in der eigenen Häuslichkeit.
- Die Demenz hat im Vergleich der häufigsten Dauerdiagnosen als einzige signifikanten Einfluss auf die eingeschätzte Betreuungsqualität.
- Ein Einfluss der Stadt-Land-Verteilung auf die Wohnsituation kann beobachtet werden und sollte in weiterer Forschung analysiert werden, um den Bedarf spezieller Betreuungsangebote zu erfassen.
